# Agreements and Discrepancies between FDA Reports and Journal Papers on Biologic Agents Approved for Rheumatoid Arthritis: *A Meta-Research Project*

**DOI:** 10.1371/journal.pone.0147556

**Published:** 2016-01-25

**Authors:** Gil Amarilyo, Daniel E. Furst, Jennifer M. P. Woo, Wen Li, Henning Bliddal, Robin Christensen, Simon Tarp

**Affiliations:** 1 Pediatric Rheumatology Unit, Schneider Children's Medical Center of Israel, Petach Tikva, and Sackler Faculty of Medicine, Tel Aviv University, Tel Aviv, Israel; 2 David Geffen School of Medicine, University of California, Los Angeles, California, United States of America; 3 Musculoskeletal Statistics Unit, The Parker Institute, Department of Rheumatology, Copenhagen University Hospital, Bispebjerg and Frederiksberg Hospital, Copenhagen, Denmark; Center for Rheumatic Diseases, INDIA

## Abstract

**Background:**

Sponsors that seek to commercialize new drugs apply to the Food and Drug Administration (FDA) which independently analyzes the raw data and reports the results on its website.

**Objectives:**

This study sought to determine if there are differences between the FDA assessments and journal reports on biologic agents developed for the treatment of rheumatoid arthritis.

**Methods:**

Available data on FDA-approved drugs were extracted from the website, and a systematic literature search was conducted to identify matching studies in peer-reviewed medical journals. Outcome measures were the American College of Rheumatology response criteria ACR20 (efficacy) and withdrawal due to adverse events (safety). As effect size odds ratios were estimated for each active trial arm vs. control arm (i.e. for both sources: FDA and journal report), followed by calculation of the ratios of the FDA and journal report odds ratios. A ratio of odds ratios not equal to 1 was categorized as a discrepancy.

**Results:**

FDA reports were available for 8 of 9 FDA-approved biologic agents for rheumatoid arthritis; all identified trials (34) except one were published in peer-reviewed journals. Overall, discrepancies were noted for 20 of the 33 evaluated trials. Differences in the apparent benefit reporting were found in 39% (24/61) pairwise comparisons and in 11 cases these were statistically significant; the FDA report showed greater benefit than the journal publication in 15 comparisons and lesser benefit in 9. Differences in the reported harms were found in 51% (28/55) pairwise comparisons and were statistically significant in 5. The “signal” in FDA reports showed a less harmful effect than the journal publication in 17 comparisons whereas a more harmful effect in 11. The differences were attributed to differences in analytic approach, patient inclusion, rounding effect, and counting discrepancies. However, no differences were categorized as critical.

**Conclusion:**

There was no empirical evidence to suggest biased estimates between the two sources. Increased and detailed transparency in publications would improve the understanding and credibility of published results. Further, the FDA report was found to be a useful source when data are missing in the published report (i.e. reporting bias).

## Introduction

Randomized controlled trials (RCTs) are part of the development program of investigational new drugs (INDs). They are usually conducted under the aegis of the sponsor (the pharmaceutical company) and are intended to provide the sponsor with the relevant data to obtain approval for marketing. In the USA, this is done by submitting a New Drug Application (NDA) to the Federal Drug Administration (FDA) [1, 2]. The NDA includes the drug’s pharmacokinetic/pharmacodynamic variables, the raw data set, copies of individual case report forms of deaths, discontinuations and serious adverse events, and statistical analyses [1–3]. It is reviewed by a panel of FDA experts (specialist physicians, statisticians, pharmacologists, and toxicologists), each of whom prepares a written evaluation of the safety and efficacy of the drug, followed by conclusions and recommendations.

After approval of the IND, the reviews are published on the FDA website (www.fda.gov) [3,4]. In some cases, the FDA commissions an independent advisory committee to review the findings. The hospitals and medical institutions that perform the clinical trials present the findings at medical conferences and subsequently publish them in peer-reviewed medical journals [3,5]. The medical community (incl. the Cochrane Collaboration) derives most of its knowledge from the published trials.

It would be reasonable to expect that the data available from the FDA reports match the results presented in the medical journals. However, studies of published premarketing trials found significant publication biases for antidepressants and, to a lesser extent, antipsychotics [6,7]. Others demonstrated that FDA findings of significant departures from good clinical practice were seldom reflected in the peer-reviewed publication [8].

The recent exponential development of new molecular entities (NMEs) has posed a challenge for the FDA partly because it required the establishment of new regulations [3]. In the field of rheumatology, the NMEs are most often biologic agents. To obtain approval to market biologic agents, sponsors submit a Biologic License Application (BLA) to the FDA [9]. Like for chemically synthesized drugs, the FDA analysis is presented on the website and the corresponding clinical trial results are published in medical journals. However, although biologic agents have revolutionized the treatment of several rheumatic diseases including rheumatoid arthritis (RA) [10], no comparisons between the FDA and the published reports have been done to date.

Meta-research is the scientific discipline that aims to evaluate and improve research practices—including thematic areas of methods, reporting, reproducibility, evaluation, and incentives [11]. The aim of the present study was to search for reporting biases and discrepancies between the FDA and medical journal reports on biologic agents for RA.

## Methods

No formal protocol is available for this current study. During the conduct of two related studies (PROSPERO: CRD42014014842, CRD42013006702) the FDA reviews were found as useful sources for data not published in the corresponding journal publication leading to the objective of this study. *A priori* we defined the applied methods for comparing results in the FDA report and the corresponding journal publications (S1 Material). The FDA-approved biologic agents for RA tested in phase 2 and 3 trials were obtained from the FDA website in April 2015. We then conducted a systematic search of three bibliographic databases through April 2015 to identify matching journal publications: the Cochrane Central Register of Controlled Trials (the Cochrane Library, latest issue), Medline, and EMBASE (S1 Table). The FDA reports and the published trial reports were then reviewed for findings on the efficacy and safety of each drug.

Efficacy was evaluated using the American College of Rheumatology response criteria (ACR20), which also served as the primary outcome measure in nearly all the RA trials. A positive ACR20 response translates into a reduction of at least 20% in the number of tender joints and swollen joints plus an improvement of at least 20% in three or more of the following five criteria: patient’s assessment of pain, patient’s assessment of disease activity, physician’s assessment of disease activity, patient’s assessment of physical function, and erythrocyte sedimentation rate or serum C-reactive protein concentration [12]. Harmful events occurring during the trial periods were evaluated by patient withdrawal due to adverse events (WdAEs) at the end of the study.

### Data collection and synthesis

The data extraction was performed by two reviewers (J.W. and W.L.) and reviewed by two others (G.A. and S.T.). Both benefits and harms were extracted from both sources, and odds ratios (ORs) were estimated for all active trial arms vs. control. The ORs of the FDA report and the corresponding published paper were compared by calculating the ratio of ORs (ROR) for all comparisons when the measure was reported in both sources, using the formula previously described by Sterne *et al*. [13]: *Exp*{ln(OR_[FDA]_)—ln(OR_[Pub]_)} in Microsoft Excel. The anticipated correlation between the two lnORs was estimated empirically across all comparisons (ACR20 r = 0.99; WdAE r = 0.93) and subsequently applied to estimate the corresponding variance of ROR as the OR measures are not mutually independent.

If a “discrepancy” was observed (ROR ≠ 1), two of the authors (G.A. and S.T.) conducted a thorough examination of the corresponding reports to determine the reason; disagreements between the authors were adjudicated by a third author (D.E.F.). In the present study, all trials are identified and coded by their FDA trial number.

## Results

**Fig 1:**
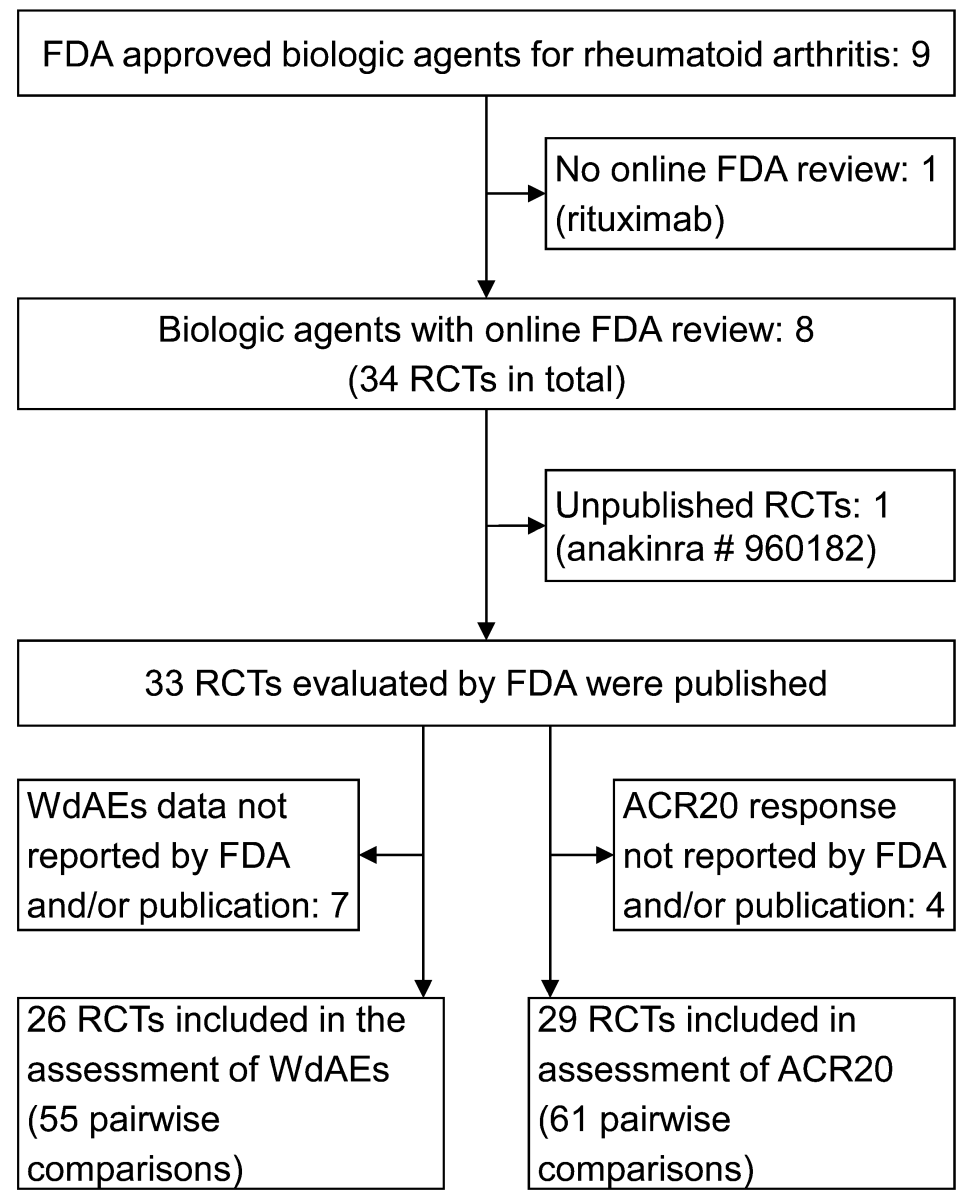
Flow diagram of retrieved and excluded randomized controlled trials. Legend: FDA = US Food and Drug Administration; RCT = randomized controlled trials; WdAE = withdrawal due to adverse events; ACR20 = American College of Rheumatology response criteria 20.

Data from 8 of the 9 FDA-approved biologic agents for RA were included in this study: abatacept, adalimumab, anakinra, certolizumab, etanercept, golimumab, infliximab, and tocilizumab. For the ninth, rituximab, the medical and statistical reviews of the randomized clinical trials were missing from the FDA website, and we were unable to retrieve them even after applying directly to the FDA. **Fig 1**illustrates a flow diagram of the retrieved and excluded articles. Overall 34 randomized controlled trials (2–6 trials per drug reviewed) were analyzed by the FDA. All but one (anakinra 960182) were published in peer-reviewed journals (S2 Table).

**Fig 2:**
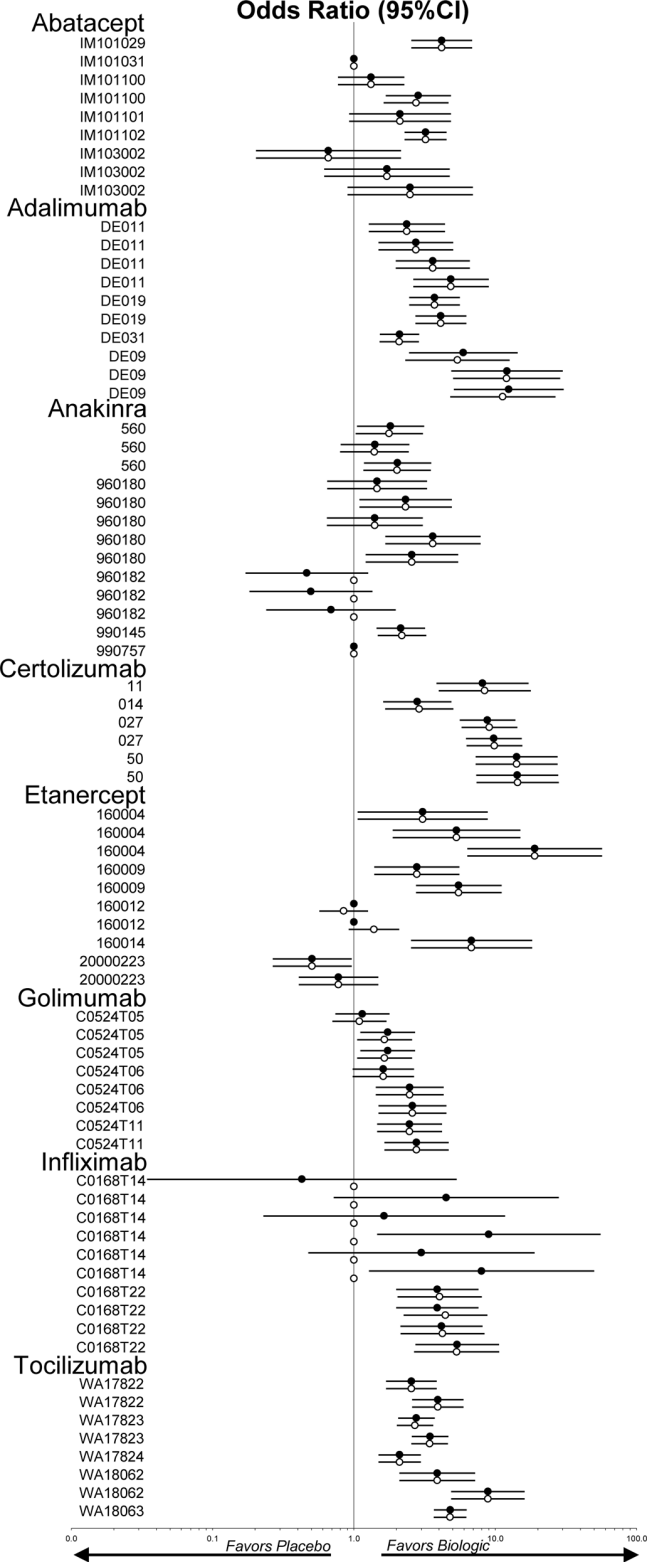
Forest plots representing the odds ratios for the ACR20 measure of the FDA review report and the journal publication. Legend: 95%CI = 95% confidence interval; Solid circles = FDA report; open circles = journal publication; ACR20 = American College of Rheumatology response criteria 20. Circles without confidence interval indicate outcome was not reported.

**Fig 3:**
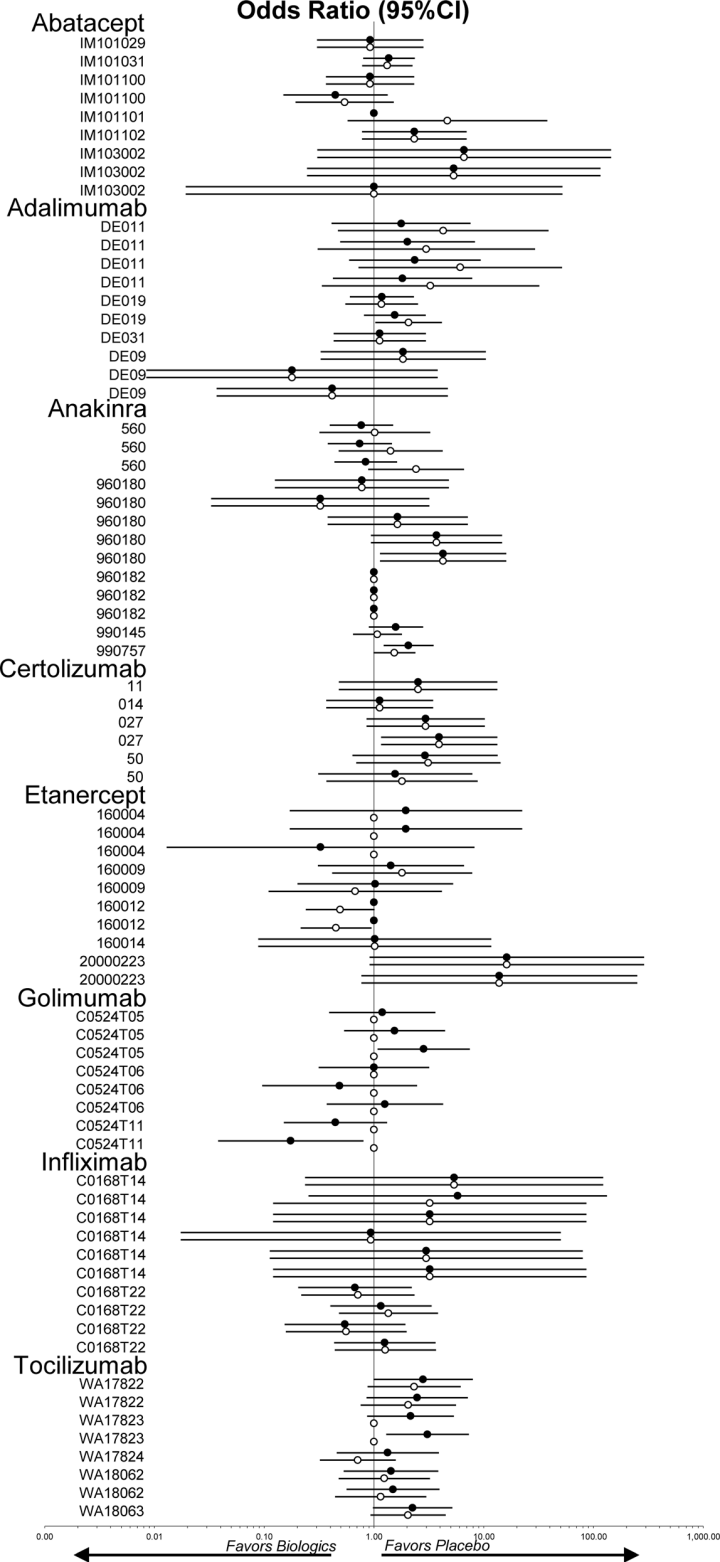
Forest plots representing the odds ratios for withdrawal due to adverse events of the FDA report and peer reviewed publication. Legend: 95%CI = 95% confidence interval; Solid circles = FDA report; open circles = journal publication. Circles without confidence interval indicate outcome was not reported.

**Figs 2 and 3**shows the forest plots that represent the odds ratios for the ACR20 and the WdAEs measures respectively on pairwise comparisons between biologics and placebo for both the FDA review analysis data and peer reviewed publication data (analyzed data available in S1 Data-Sheet).

### Comparison of the benefit assessments

**Fig 4:**
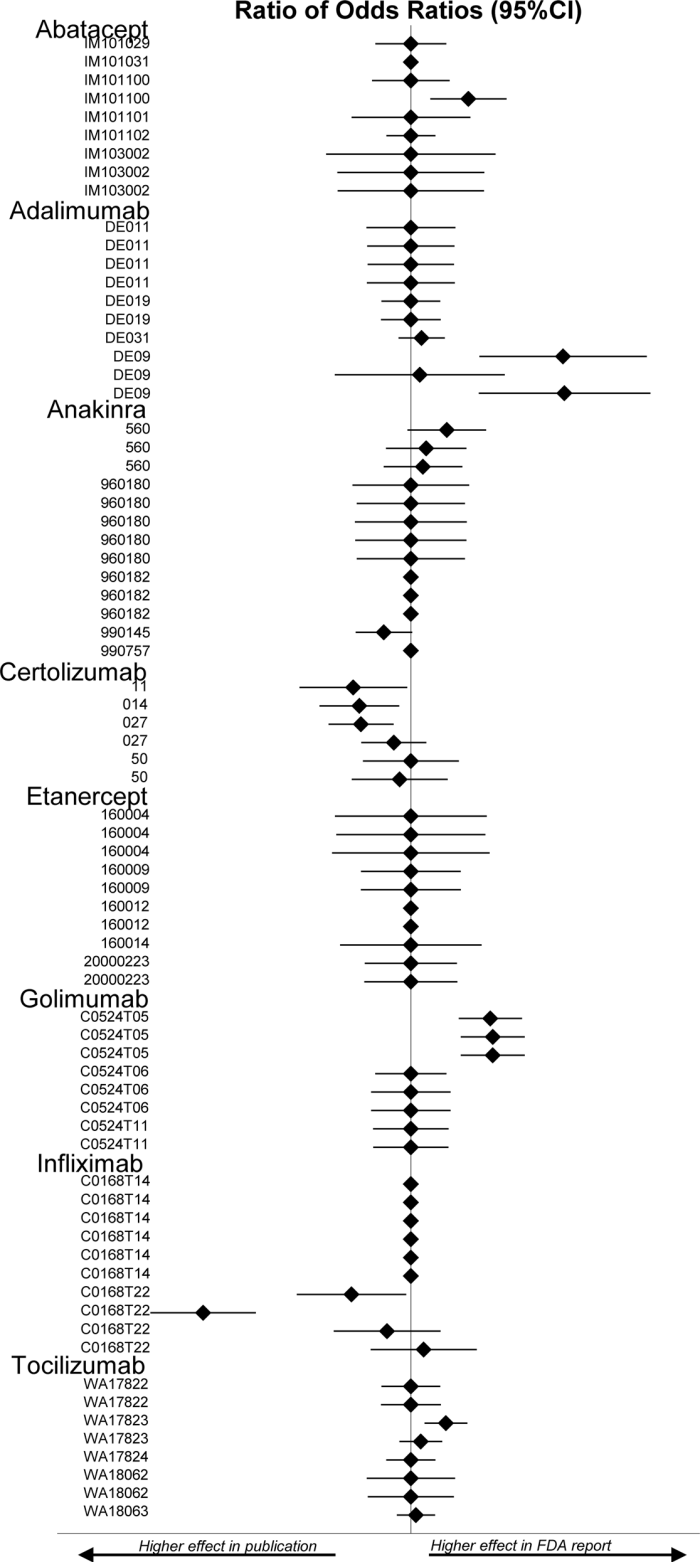
Forest plots representing the ratio of odds ratios for the ACR20 measure between FDA review analysis data and peer reviewed publication data. Legend: ACR20 = American College of Rheumatology response criteria 20; 95%CI = 95% confidence interval. Diamonds without confidence interval indicate outcome was not reported in one or both sources.

Of the 33 trials published in peer-reviewed journals, 4 were excluded from the ACR20 analysis because pertinent data were missing from the FDA website (etanercept 160012), in the published report (infliximab C0168T14), or from both the FDA website and the published report (abatacept IM101031 and anakinra 990757). The 29 remaining trials yielded a total of 61 comparisons (several trials had a multiple trial-arm design) **Fig 4**. As summarized in **Table 1**disagreement was found in 24 (39%) of the 61 comparisons, including 11 in which the difference was statistically significant (p<0.05) (S3 Table).

**Table 1:** Summary of discrepancies between FDA and peer-reviewed journal reports.

Drug	No. of analyzed RCTs	No. of analyzed arms (vs. placebo)	Matched	Favors FDA	Favors publication
**ACR20**					
Abatacept	5	8	7	1	-
Adalimumab	4	10	6	4	-
Anakinra	3	9	5	3	1
Certolizumab	4	6	1	-	5
Etanercept	4	8	8	-	-
Golimumab	3	8	5	3	-
Infliximab	1	4	-	1	3
Tocilizumab	5	8	5	3	-
**Total**	29	61 (100%)	37 (61%)	15 (24%)	9 (15%)
**WdAEs**					
Abatacept	5	8	6	1	1
Adalimumab	4	10	6	4	-
Anakinra	4	10	5	3	2
Certolizumab	4	6	2	4	-
Etanercept	3	5	3	1	1
Golimumab	-	-	-	-	-
Infliximab	2	10	5	4	1
Tocilizumab	4	6	-	-	6
**Total**	26	55 (100%)	27 (49%)	17 (31%)	11 (20%)

RCT = randomized controlled trial, No. = number of patients, ACR20 = American College of Rheumatology 20, WdAEs = withdrawal of patients due to adverse events, FDA = Food and Drug Administration.

In 9 comparisons, the journal report showed greater efficacy than the FDA report, and in 15 comparisons, the FDA report showed better efficacy. As summarized in S4 Table, there were several reasons for these discrepancies: i) Difference in analytic approach (9 comparisons)–one source used the ITT population whereas the other used the modified ITT; ii) Difference in patient included in the analysis (6 comparisons)—the FDA report excluded patients from disbarred sites (adalimumab SE09 and DE31) or patients who were missing ACR20 assessments. For example, in the certolizumab trial (27), subjects with no ACR20 data were included in the denominator in the published report but not in the FDA report. When the FDA conducted a further calculation using the non-responder imputation method, as in the published study, the results were similar in both reports; iii) Rounding effect (6 comparisons)—the published report described the data in percent whereas the FDA report described the data by number of responders; iv) Counting discrepancies (3 comparisons)–The FDA and published reports differed in the number of patients analyzed or number of patients with a particular finding. In comparisons included in this category, we could not find a reasonable explanation for the discrepancy.

### Comparison of harm assessments

**Fig 5:**
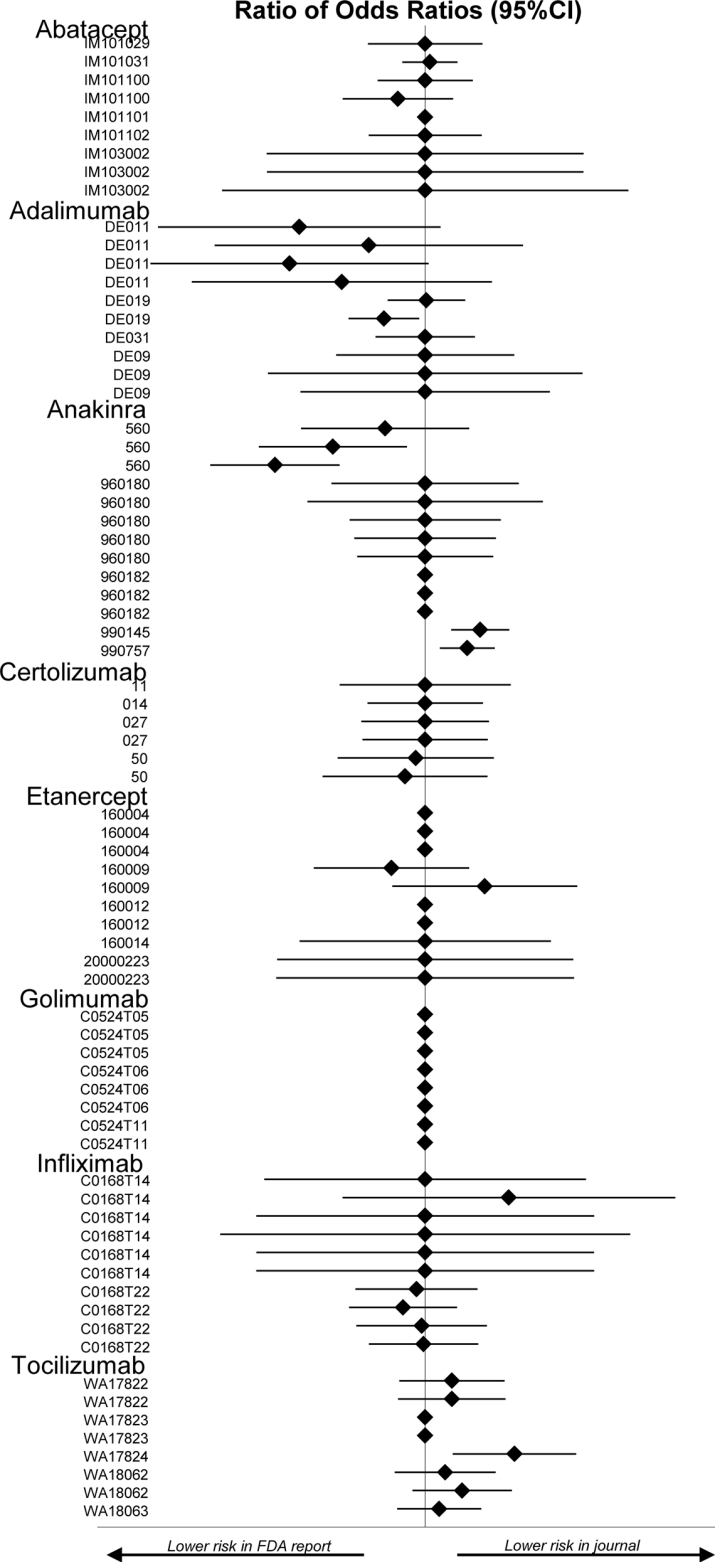
Forest plots representing the ratio of odds ratios for the WdAE measure on pairwise comparisons between FDA review analysis data and peer reviewed publication data. Legend: WdAE = withdrawal due to adverse events; 95%CI = 95% confidence interval. Diamonds without confidence interval indicate outcome was not reported in one or both sources.

Of the 33 trials published in peer-reviewed journals, 7 were excluded from the WdAEs analysis because pertinent data were missing from the FDA reports (etanercept 160004; etanercept 160012; abatacept IM101101, tocilizumab WA17823) or the published report (golimumab C0524T05, C0524T06 and C0524T11). The 26 remaining trials yielded a total of 55 comparisons **Fig 5**. As summarized in Table 1 disagreement was found in 28 of the 55 comparisons including 5 in which the difference was statistically significant (p<0.05) (S4 Table).

In 11 comparisons, the journal report showed higher safety (lower WdAE estimates) than the FDA report, and in 17 comparisons, the FDA report showed higher safety than the journal report. As summarized in S3 Table, 20 discrepancies were attributed to counting differences (no clear explanation). The other reasons were differences in patient inclusion (6 comparisons) and in analytic approach (2 comparisons).

## Discussion

The present study compared the data on new biologic drugs for RA published on the FDA website and in peer-reviewed medical journals. In addition to thorough and independent analysis of raw data from RCT's, the FDA reviews offer a unique source of information when preforming meta-analysis for missing data in the journal publication. Discrepancies in outcome were noted in a substantial proportion of pairwise comparisons in terms of benefit measures (ACR20 responses, 39%) and harms (WdAEs, 51%). However, closer inspection revealed that many of the discrepancies in the ACR20 measure were caused merely by rounding effects or inclusion/ exclusion of patients from the analysis. Moreover, in 63% of the pairwise comparisons, the FDA analysis favored the test medication relative to the peer-reviewed publication, which weighs strongly against reporting bias toward positive efficacy outcomes.

For WdAEs, conclusions regarding differences between the sources and reporting bias were more complex. In almost half the comparisons with discrepant findings (11/28), the published reports showed higher safety than the FDA reports. However, 9 of the differences were explained by counting discrepancies, i.e., we could not find a reasonable explanation for them, and only one-third were statistically significant. Usually, there were differences of 1–2 patients between the FDA and the journal reports, but in some cases, the counting discrepancies were larger. For example, in the anakinra 560 trial, major discrepancies were found in all arms: for anakinra 30 mg, the FDA reported 19 events and the published study, 6 events; for anakinra 75 mg, 18 and 8 events, respectively; for anakinra 150 mg, 20 and 13 events, respectively; and for placebo, 24 and 6 events, respectively. This raises the question of reporting bias toward more favorable safety results, particularly because the counting discrepancies could not be explained (which was true even for anakinra). On the other hand, in 61% of the discrepancies, fewer WdAEs were found in the FDA analyses, indicating no such bias.

In some instances, the analytic approach differed between the FDA and the published reports, mainly because one analysis used a strict ITT population and other datasets were based on a modified ITT (e.g., treated) population. It is noteworthy that the FDA itself was not consistent in its preference for one or the other approach among the analyzed trials. Indeed, in some trials, a modified ITT analysis was done first, followed by additional analyses for the primary endpoint using the strict ITT subset with a non-responder imputation for missing data. The FDA has written guidelines but not overly rigid requirements for the design or conduct of clinical trials and the specific data that must be collected [14]. We believe requiring transparency, consistency, and detailed completeness in study analyses would help to minimize discrepancies of this type and avoid bias.

Unlike similar studies in other fields of medicine [6,7,15], the discrepancies between the FDA and the published reports for the outcome measures evaluated were not sufficiently large to change the main efficacy and safety conclusions of the trial. However, we believe that after its summaries are made available to the public, the FDA could notify authors of the trial studies about discrepancies in the results. This would encourage authors to review their data and lead to greater transparency and consistency in the analyses.

Finally, trials that are conducted but not published are a matter of concern, especially if they report negative results and/or do not achieve their primary outcomes. Indeed, one low-dose (2.5, 10, 30 mg/day) short-term (12 week) anakinra monotherapy trial (no. 960182) that was included in the BLA along with its open-label extension (no. 970102) were never published. In this study, there was no significant difference in ACR20 response or other efficacy endpoint between any of the anakinra arms and placebo at 12 weeks. On further examination to determine if these results were due to baseline covariates, subject/disease characteristics, or potential safety issues, no explanation for the lack of effect was found [16]. According to the FDA Arthritis Drugs Advisory Committee Briefing Package Report for Anakinra, the unpublished study provided support for utilization of a higher dose of anakinra in the treatment of subjects with RA [16].

Our study has two main limitations. First, we did not include FDA-approved rituximab because no review (medical or statistical) was available on the FDA website, and we were unable to access the data even after applying directly to the FDA authorities. Second, we examined a single efficacy measure and a single safety measure. These were used mainly because they were the outcome measures that were common to most of the studies and could therefore be analyzed across studies.

In conclusion, pairwise comparisons and FDA reviews of new biologic agents for RA and the corresponding published clinical trials show a discrepancy in efficacy in about one-half of cases and in safety in about one-third of cases. Many factors may account for these differences. Although some of the differences were statistically significant, they did not affect the overall conclusions. Importantly, given that the medical community tends to derive most of its knowledge from peer-reviewed journals, there was no empirical evidence to suggest reporting bias. Increased detailed description and transparency in publications would increase the credibility of published results. Further, the FDA report was found to be a useful source when data are missing in the published report (i.e. reporting bias).

## Supporting Information

S1 Data-Sheet Full dataset. (XLSX)

S1 Material A priori defined methods. (PDF)

S2 Material PRISMA checklist. (DOCX)

S1 Table Search Strategies. (DOCX)

S2 Table Reference list of the 33 published RCTs. (DOCX)

S3 Table Types of ACR20 discrepancies, where discrepancies were observed between data reviewed by the FDA and data published in peer-review journals. (DOCX)

S4 Table Types of “withdrawal due to adverse events” discrepancies, where discrepancies were observed between data reviewed by the FDA and data published in peer-reviewed journals. (DOCX)
