# Supplementary material for: Agreements and Discrepancies between FDA Reports and Journal Papers on Biologic Agents Approved for Rheumatoid Arthritis: A Meta-Research Project
Source: PLoS One. 2016 Jan 25;11(1):e0147556. doi: 10.1371/journal.pone.0147556 (PMC4725722; doi:10.1371/journal.pone.0147556)
Supplement: S1 Material — (PDF) [file pone.0147556.s002.pdf]

DISCREPANCY BETWEEN THE FDA REPORT AND THE PUBLISHED PAPER

Robin Christensen
Musculoskeletal Statistics Unit, The Parker Institute
Copenhagen, Denmark.

For both types of publications, data will be collected in the following 2x2 contingency table format:

GROUP	RESPONSE	NO RESPONSE	TOTAL
Intervention	a	b	N_I
Comparator	c	d	N_C

Effect measure Odds Ratio and its corresponding variance:

$$OR = (a/b) / (c/d)$$

$$Var(\ln OR) = 1/a + 1/b + 1/c + 1/d$$

These parameter estimates are calculated independent for each type of publication:

OR_{FDA} & $Var(\ln OR_{FDA})$ & $SE(\ln OR_{FDA})$

OR_{Pub} & $Var(\ln OR_{Pub})$ & $SE(\ln OR_{Pub})$

The Ratio of Odds Ratios is calculated as:

$$ROR = Exp(\ln(OR_{FDA}) - \ln(OR_{Pub}))$$

$$Var(\ln ROR) = Var(\ln OR_{FDA}) + Var(\ln OR_{Pub}) - 2*r*SE(\ln OR_{FDA})*SE(\ln OR_{Pub})$$

The anticipated correlation (r) between the two ln*OR* measures will be estimated empirically using a Pearson correlation coefficient across all the pairwise comparisons.

Statistical inference on the study level.

The Standard Error (SE) for the ln*ROR*:

$SE(\ln ROR) = Var(\ln ROR)^{1/2}$

Statistical test:

$Z = \ln ROR / SE(\ln ROR)$
If abs(Z) > 1.96 then P-value < 0.05

95% Confidence Intervals (95% CIs) for *ROR*:

Lower 95% Confidence Limit: $Exp(\ln ROR - 1.96 * SE(\ln ROR))$
Upper 95% Confidence Limit: $Exp(\ln ROR + 1.96 * SE(\ln ROR))$

For sensitivity these analyses will also be tested with alternative r-correlation coefficients:

Sens (#1): $r_{\#1} = 0.750$
Sens (#2): $r_{\#2} = 0.999$
