# Supplementary material for: Agreements and Discrepancies between FDA Reports and Journal Papers on Biologic Agents Approved for Rheumatoid Arthritis: A Meta-Research Project
Source: PLoS One. 2016 Jan 25;11(1):e0147556. doi: 10.1371/journal.pone.0147556 (PMC4725722; doi:10.1371/journal.pone.0147556)
Supplement: S1 Table — (DOCX) [file pone.0147556.s004.docx]

# S1 Table: Search Strategies

| PubMed search strategy |
| --- |
| (((("Receptors, Tumor Necrosis Factor"[nm] OR TNFR:Fc OR "TNFR-Fc fusion protein"[Supplementary Concept] OR "TNFR-Fc fusion protein"[All Fields] OR "etanercept"[All Fields] OR "enbrel"[All Fields]) OR ("infliximab"[Supplementary Concept] OR "infliximab"[All Fields] OR "remicade"[All Fields] OR "mab ca2"[All Fields] OR "monoclonal antibody ca2"[All Fields]) OR ("adalimumab"[Supplementary Concept] OR "adalimumab"[All Fields] OR "humira"[All Fields]) OR ("interleukin 1 receptor antagonist protein"[MeSH Terms] OR "interleukin 1 receptor antagonist protein"[All Fields] OR "anakinra"[All Fields] OR "kineret"[All Fields] OR "antril"[All Fields]) OR ("abatacept"[Supplementary Concept] OR "abatacept"[All Fields]) OR CTLA4Ig[All Fields] OR "orencia"[All Fields]) OR ("rituximab"[Supplementary Concept] OR "rituximab"[All Fields] OR "rituxan"[All Fields] OR "idec c2b8"[All Fields]) OR ("golimumab"[All Fields] OR "golimumab"[Supplementary Concept] OR "simponi"[All Fields] OR "cnto-148"[All Fields] OR ("cnto"[All Fields] AND "148"[All Fields])) OR ("tocilizumab"[All Fields] OR "tocilizumab"[Supplementary Concept] OR "atlizumab"[All Fields] OR "actemra"[All Fields]) OR ("certolizumab"[All Fields] OR "certolizumab pegol"[Supplementary Concept] OR "CDP870"[All Fields] OR ("cdp"[All Fields] AND "870"[All Fields]) OR "cimzia"[All Fields]) OR ("tofacitinib"[Supplementary Concept] OR "tofacitinib"[All Fields]) OR ("Antibodies, Monoclonal"[Mesh] OR "Monokines"[Mesh] OR "Receptors, Interleukin-1"[Mesh] OR "Receptors, Interleukin-6"[Mesh])) AND ("Randomized Controlled Trial"[ptyp] OR "Controlled Clinical Trial"[ptyp] OR "Multicenter Study"[ptyp] OR "randomized"[tiab] OR "randomised"[tiab] OR "placebo"[tiab] OR "randomly"[tiab] OR "trial"[tiab] OR randomized controlled trials[mh] OR random allocation[mh] OR double-blind method[mh] OR single-blind method[mh]) AND ("Arthritis, Rheumatoid"[MeSH Terms] OR (Rheumatoid[text word] AND arthriti*[text word]))) NOT (animals[mh] NOT human[mh]) |
| The Cochrane Central Register of Controlled Trials (CENTRAL) search strategy |
| \| ID \| Search \| \| --- \| --- \| \| #1 \| MeSH descriptor: [Recombinant Fusion Proteins] explode all trees \| \| #2 \| MeSH descriptor: [Antibodies, Monoclonal] explode all trees \| \| #3 \| MeSH descriptor: [Receptors, Tumor Necrosis Factor] explode all trees \| \| #4 \| MeSH descriptor: [Receptors, Interleukin-1] explode all trees \| \| #5 \| MeSH descriptor: [Receptors, Interleukin-6] explode all trees \| \| #6 \| MeSH descriptor: [Monokines] explode all trees \| \| #7 \| monoclonal antibody ca2 \| \| #8 \| TNFR-Fc fusion protein \| \| #9 \| MeSH descriptor: [Interleukin 1 Receptor Antagonist Protein] explode all trees \| \| #10 \| etanercept \| \| #11 \| enbrel \| \| #12 \| infliximab \| \| #13 \| remicade \| \| #14 \| adalimumab \| \| #15 \| humira \| \| #16 \| D2E7 \| \| #17 \| anakinra \| \| #18 \| kineret \| \| #19 \| antril \| \| #20 \| abatacept \| \| #21 \| CTLA4Ig \| \| #22 \| orencia \| \| #23 \| rituximab \| \| #24 \| rituxan \| \| #25 \| idec c2b8 \| \| #26 \| golimumab \| \| #27 \| simponi \| \| #28 \| cnto-148 \| \| #29 \| tocilizumab \| \| #30 \| atlizumab \| \| #31 \| actemra \| \| #32 \| roactemra \| \| #33 \| certolizumab \| \| #34 \| CDP870 \| \| #35 \| cimzia \| \| #36 \| "TNFR:Fc":ti,ab,kw (Word variations have been searched) \| \| #37 \| tofacitinib:ti,ab,kw (Word variations have been searched) \| \| #38 \| MeSH descriptor: [Janus Kinases] explode all trees \| \| #39 \| Xeljanz:ti,ab,kw (Word variations have been searched) \| \| #40 \| #1 or #2 or #3 or #4 or #5 or #6 or #7 or #8 or #9 or #10 or #11 or #12 or #13 or #14 or #15 or #16 or #17 or #18 or #19 or #20 or #21 or #22 or #23 or #24 or #25 or #26 or #27 or #28 or #29 or #30 or #31 or #32 or #33 or #34 or #35 or #36 or #37 or #38 or #39 \| \| #41 \| MeSH descriptor: [Arthritis, Rheumatoid] explode all trees \| \| #42 \| Rheumatoid:ti or Rheumatoid:ab (Word variations have been searched) \| \| #43 \| arthriti*:ti or arthriti*:ab (Word variations have been searched) \| \| #44 \| #42 and #43 \| \| #45 \| #41 or #44 \| \| #46 \| #40 and #45 in Trials \| |
| EMBASE search strategy |
| \| 1 \| abatacept.mp. \| \| --- \| --- \| \| 2 \| adalimumab.mp. \| \| 3 \| certolizumab.mp. \| \| 4 \| etanercept.mp. \| \| 5 \| CDP870.mp. \| \| 6 \| golimumab.mp. \| \| 7 \| infliximab.mp. \| \| 8 \| rituximab.mp. \| \| 9 \| tocilizumab.mp. \| \| 10 \| humira.mp. \| \| 11 \| trudexa.mp. \| \| 12 \| orencia.mp. \| \| 13 \| cimzia.mp. \| \| 14 \| enbrel.mp. \| \| 15 \| simponi.mp. \| \| 16 \| rituxan.mp. \| \| 17 \| mabthera.mp. \| \| 18 \| actemra.mp. \| \| 19 \| RoActemra.mp. \| \| 20 \| monoclonal antibodies.mp. or exp Antibodies, Monoclonal/ \| \| 21 \| exp Monokines/ \| \| 22 \| exp Receptors, Interleukin-1/ \| \| 23 \| exp Receptors, Interleukin-6/ \| \| 24 \| exp Polyethylene Glycols/ \| \| 25 \| exp Immunoglobulin G/ \| \| 26 \| exp Immunoconjugates/ \| \| 27 \| immunoglobulin fab fragments.mp. or exp Immunoglobulin Fab Fragments/ \| \| 28 \| t-lymphocytes.mp. or exp T-Lymphocytes/ \| \| 29 \| exp tumor necrosis factor inhibitor/ \| \| 30 \| exp interleukin 1 receptor blocking agent/ \| \| 31 \| D2E7.mp. \| \| 32 \| anakinra.mp. \| \| 33 \| kineret.mp. \| \| 34 \| antril.mp. \| \| 35 \| CTLA4Ig.mp. \| \| 36 \| idec c2b8.mp. \| \| 37 \| cnto-148.mp. \| \| 38 \| atlizumab.mp. \| \| 39 \| tofacitinib.mp. \| \| 40 \| exp Janus kinase inhibitor/ \| \| 41 \| *tumor necrosis factor receptor/dt [Drug Therapy] \| \| 42 \| or/1-41 \| \| 43 \| exp Random Allocation/ \| \| 44 \| exp Single-Blind Method/ \| \| 45 \| exp Double-Blind Method/ \| \| 46 \| Placebo.mp. \| \| 47 \| Randomi?ed controlled trial$.mp. \| \| 48 \| rct.mp. \| \| 49 \| Random allocation.mp. \| \| 50 \| Randomly allocated.mp. \| \| 51 \| Allocated randomly.mp. \| \| 52 \| (allocated adj2 random).mp. \| \| 53 \| Single blind$.mp. \| \| 54 \| Double blind$.mp. \| \| 55 \| ((treble or triple) adj blind$).mp. \| \| 56 \| Placebo$.mp. \| \| 57 \| or/43-56 \| \| 58 \| rheumatoid.ti,ab. \| \| 59 \| *rheumatoid arthritis/ \| \| 60 \| 58 or 59 \| \| 61 \| 42 and 57 and 60 \| \| 62 \| limit 61 to (book or book series or conference abstract or conference paper or conference proceeding or "conference review") \| \| 63 \| 61 not 62 \| |
