# Supplementary material for: Agreements and Discrepancies between FDA Reports and Journal Papers on Biologic Agents Approved for Rheumatoid Arthritis: A Meta-Research Project
Source: PLoS One. 2016 Jan 25;11(1):e0147556. doi: 10.1371/journal.pone.0147556 (PMC4725722; doi:10.1371/journal.pone.0147556)
Supplement: S2 Table — (DOCX) [file pone.0147556.s005.docx]

# S2 Table: Reference list of the 33 published RCTs.

| **RCT no.** | **Biological agent** | **Manufacturer** | **Reference** |
| --- | --- | --- | --- |
| IM101029 | Abatacept | Bristol-Myers Squibb Company | Genovese et al. N Engl J Med. 2005 |
| IM101031 |  |  | Weinblatt et al. Arthritis Rheum. 2006 |
| IM101100 |  |  | Kremer et al. N Engl J Med. 2003 |
| IM101101 |  |  | Weinblatt et al. Ann Rheum Dis. 2007 |
| IM101102 |  |  | Kremer et al. Ann Intern Med. 2006 |
| IM103002 |  |  | Moreland et al. Arthritis Rheum. 2002 |
| DE011 | Adalimumab | AbbVie | van de Putte et al. Ann Rheum Dis. 2004 |
| DE019 |  |  | Keystone et al. Arthritis Rheum. 2004 |
| DE031 |  |  | Furst et al. J Rheumatol. 2003 |
| DE09 |  |  | Weinblatt et al. Arthritis Rheum. 2003 |
| 560 | Anakinra | Sobi | Bresnihan et al. Arthritis Rheum. 1998 |
| 960180 |  |  | Cohen et al. Arthritis Rheum. 2002 |
| 990145 |  |  | Cohen et al. Ann Rheum Dis. 2004 |
| 990757 |  |  | Fleischmann et al. Arthritis Rheum. 2003 |
| 11 | Certolizumab | UCB | Fleischmann et al. Ann Rheum Dis. 2009 |
| 014 |  |  | Choy et al. Rheumatology (Oxford) 2012 |
| 027 |  |  | Keystone et al. Arthritis Rheum. 2008 |
| 50 |  |  | Smolen et al. Ann Rheum Dis. 2009 |
| 160004 | Etanercept | Pfizer | Moreland et al. N Engl J Med. 1997 |
| 160009 |  |  | Moreland et al. Ann Intern Med 1999 |
| 160012 |  |  | Bathon et al. N Engl J Med. 2000 |
| 160014 |  |  | Weinblat et al. N Engl J Med. 1999 |
| 20000223 |  |  | Genovese et al. Arthritis Rheum. 2004 |
| C0524T05 | Golimumab | Janssen Biotech. | Emery et al. Arthritis Rheum. 2009 |
| C0524T06 |  |  | Keystone et al. Ann Rheum Dis. 2011 |
| C0524T11 |  |  | Smolen et al. Lancet 2009 |
| C0168T14 | Infliximab | Janssen Biotech. | Maini et al. Arthritis Rheum. 1998 |
| C0168T22 |  |  | Lipsky et al. N Engl J Med. 2000 |
| WA17822 | Tocilizumab | Genentech | Smolen et al. Lancet 2008 |
| WA17823 |  |  | Kremer et al. Arthritis Rheum. 2011 |
| WA17824 |  |  | Jones et al. Ann Rheum Dis. 2010 |
| WA18062 |  |  | Emery et al. Ann Rheum Dis. 2008 |
| WA18063 |  |  | Genovese et al. Arthritis Rheum. 2008 |
