# Supplementary material for: Agreements and Discrepancies between FDA Reports and Journal Papers on Biologic Agents Approved for Rheumatoid Arthritis: A Meta-Research Project
Source: PLoS One. 2016 Jan 25;11(1):e0147556. doi: 10.1371/journal.pone.0147556 (PMC4725722; doi:10.1371/journal.pone.0147556)
Supplement: S3 Table — (DOCX) [file pone.0147556.s006.docx]

**S3 Table: Types of ACR20 discrepancies, where discrepancies were observed between data reviewed by the FDA and data published in peer-review journals**

| **RCT no.** | **Arm (vs. placebo)** | **Favors** | **ROR** | **P value** | **Analysis** | **Discrepancy type** |
| --- | --- | --- | --- | --- | --- | --- |
| IM101100 | Abatacept 10 mg | FDA | 1.04 | 0.003 | 60% responders in publication vs. 61% in FDA report. | Rounding effect |
| DE031 | Adalimumab 40 mg | FDA | 1.01 | 0.36 | One investigator at site #7 was in the process of being disbarred, and all subjects (6) enrolled at the site excluded from the FDA analysis. | Patient inclusion |
| DE09 | Adalimumab 20 mg | FDA | 1.10 | <0.001 | One investigator at site #7 was in the process of being disbarred, and all subjects (11) enrolled at the site were excluded from the FDA analysis. | Patient inclusion |
|  | Adalimumab 40 mg | FDA | 1.01 | 0.837 |  |  |
|  | Adalimumab 80 mg | FDA | 1.10 | <0.001 |  |  |
| 560 | Anakinra 30 | FDA | 1.02 | 0.072 | Analyzed population was smaller in the publication because the FDA analysis was based on the intention to treat (ITT) population. | Analytic approach |
|  | Anakinra 75 | FDA | 1.01 | 0.455 |  |  |
|  | Anakinra 150 | FDA | 1.01 | 0.544 |  |  |
| 990145 | Anakinra 100 | Journal | 0.98 | 0.061 | Response rate in publication was 38% of 250. Exact number of responders is not mentioned but can be calculated (95). FDA mentions 94/250 responders, which is 37.6%. Differences may be due to rounding 37.6% to 38%. | Rounding effect |
| 11 | Certolizumab 400 | Journal | 0.96 | 0.036 | In publication, ACR20 was presented only as a rounded percentage (45.5%). In the FDA report, both rounded percentage (45%) and number of responders were reported. | Rounding effect |
| 14 | Certolizumab 400 | Journal | 0.97 | 0.011 | In the FDA report, 56/124 (45%) achieved ACR20 at week 24 vs. 45.9% (calculated as 57/124) reported in publication. | Counting discrepancy |
| 27 | Certolizumab 200 | Journal | 0.99 | 0.536 | The FDA report did not include 5 patients from the CER200 arm, 2 patients from the CER400 arm, and 1 patient from the placebo arm because "the biologic license application (BLA) did not include subjects who were missing all ACR assessments". However, the FDA calculated the results with all missing set to non-responder (not shown in the report), and the results were consistent. | Counting discrepancy |
|  | Certolizumab 400 | Journal | 1.0 | 0.739 |  |  |
| **RCT no.** | **Arm (vs. placebo)** | **Favors** | **ROR** | **P value** | **Analysis** | **Discrepancy type** |
| 50 | Certolizumab 400 | Journal | 0.99 | 0.647 | The FDA report notes that: "For the ACR response variables, the sponsor did not include one subject in the Cimizia 400mg group due to all ACR measures being missing." In the publication, there are 142/246 responders in the Cimzia 400 arm and in the FDA report, 141/245. | Counting discrepancy |
| C0524T05 | Golimumab 100 mg | FDA | 1.05 | <0.001 | In the FDA report, ACR20 was presented as a rounded percentage. In the publication, both the exact percentage and the number of responders were reported. | Rounding effect |
|  | Golimumab 50 mg+MTX | FDA | 1.05 | <0.001 |  |  |
|  | Golimumab 100 mg+MTX | FDA | 1.05 | <0.001 |  |  |
| C0168T22 | Infliximab 3 mg at week 4 | Journal | 0.88 | <0.001 | Three different populations were used for the different analyses in the publication. Demographics were determined using the randomized populations; efficacy at week 30 was determined using only subjects evaluated at week 30, and the safety analysis accounted for deviations in the protocol. In the FDA report, analyses were based on the ITT population. | Analytic approach |
|  | Infliximab 3 mg at week 8 | Journal | 0.96 | 0.033 |  |  |
|  | Infliximab 10 mg at week 4 | FDA | 1.01 | 0.633 |  |  |
|  | Infliximab 10 mg at week 8 | Journal | 0.99 | 0.383 |  |  |
| WA17823 | Tocilizumab 4 mg | FDA | 1.02 | 0.001 | FDA report used randomized populations whereas the publication used the ITT population. | Analytic approach |
|  | Tocilizumab 8 mg | FDA | 1.01 | 0.364 |  |  |
| WA18063 | Tocilizumab 8 mg | FDA | 1.00 | 0.599 | The publication stated that "Of the 805 patients randomized to receive tocilizumab and the 415 patients randomized to the control group, 803 and 413, respectively, were included in the ITT population." Although the FDA report states that it used an ITT approach, in fact, the total randomized population was analyzed. This did not affect the response percentages (61% for tocilizumab vs. 25% for placebo). | Counting discrepancy |

RCT-randomized controlled trial, No.-number of patients, ACR20-American College of Rheumatology 20, FDA - Food and Drug Administration
