# Supplementary material for: Agreements and Discrepancies between FDA Reports and Journal Papers on Biologic Agents Approved for Rheumatoid Arthritis: A Meta-Research Project
Source: PLoS One. 2016 Jan 25;11(1):e0147556. doi: 10.1371/journal.pone.0147556 (PMC4725722; doi:10.1371/journal.pone.0147556)
Supplement: S4 Table — (DOCX) [file pone.0147556.s007.docx]

**S4 Table: Types of WdAEs discrepancies, where discrepancies were observed between data reviewed by the FDA and data published in peer-reviewed journals**

| **RCT no.** | **Arm (vs. placebo)** | **Favors** | **ROR** | **P value** | **Analysis** | **Discrepancy type** |
| --- | --- | --- | --- | --- | --- | --- |
| IM101031 | Abatacept 10 mg | Journal | 1.03 | 0.738 | 19/51 withdrew due to AEs in journal report vs. 20/52 in FDA report. | Counting discrepancy |
| IM101100 | Abatacept 10 mg | FDA | 0.83 | 0.333 | 6 withdrew due to AEs in journal report vs. 5 in FDA report. | Counting discrepancy |
| DE011 | Adalimumab 20 mg_1 | FDA | 0.67 | 0.473 | Discrepancies between journal and FDA reports in all arms. FDA report data are found in the FDA statistical review table 33 (AE leading to permanent withdrawal**)** | Counting discrepancy |
|  | Adalimumab 20 mg_2 | FDA | 0.41 | 0.081 |  |  |
|  | Adalimumab 40 mg_1 | FDA | 0.56 | 0.275 |  |  |
|  | Adalimumab 40 mg_2 | FDA | 0.39 | 0.056 |  |  |
| 560 | Anakinra 30 | FDA | 0.75 | 0.35 | Major discrepancies between journal and FDA reports in all arms. For 30 mg, 75 mg, 150 mg, and placebo: 6, 8, 13, and 6 patients withdrew due to AEs in journal report vs. 19, 18, 20, and 24 in FDA report, respectively. | Counting discrepancy |
|  | Anakinra 75 | FDA | 0.52 | 0.014 |  |  |
|  | Anakinra 150 | FDA | 0.35 | <0.001 |  |  |
| 990145 | Anakinra 100 | Journal | 1.47 | <0.001 | Discrepancies between journal and FDA reports in all arms. For 100 mg and placebo: 33 and 22 patients withdrew due to AEs in journal report vs. 35 and 33 in FDA report, respectively. | Counting discrepancy |
| 990757 | Anakinra 100 | Journal | 1.34 | 0.002 | Discrepancies between journal and FDA reports in all arms. For 100 mg and placebo: 130 and 17 patients withdrew due to AEs in journal report vs. 150 and 26 in FDA report, respectively. | Counting discrepancy |
| 27 | Certolizumab 200 | FDA | 1.00 | 0.991 | Total patients in FDA safety analysis differ between for CER200 (n=392) and CER 400 (n=389). Total patients also differ from journal efficacy analysis (n=393 and 390, respectively). | Counting discrepancy |
|  | Certolizumab 400 | FDA | 1.00 | 0.991 |  |  |
| 50 | Certolizumab 200 | FDA | 0.94 | 0.812 | In journal report, 2 patients in the placebo group received certolizumab pegol 200 mg and were included in the certolizumab pegol 200 mg group for safety evaluations. | Patient inclusion |
|  | Certolizumab 400 | FDA | 0.87 | 0.630 |  |  |
| 160009 | Etanercept 10 mg | FDA | 0.79 | 0.394 | 5 patients withdrew due to AEs in journal report vs. 4 in FDA report. | Counting discrepancy |
|  | Etanercept 25 mg | Journal | 1.52 | 0.206 | 2 patients withdrew due to AEs in journal report vs. 3 in FDA report. | Counting discrepancy |
| C0168T14 | Infliximab 1 mg+MTX | Journal | 1.80 | 0.324 | 1 patient withdrew due to AEs in journal report vs. 2 in FDA report. | Counting discrepancy |

| **RCT no.** | **Arm (vs. placebo)** | **Favors** | **ROR** | **P value** | **Analysis** | **Discrepancy type** |
| --- | --- | --- | --- | --- | --- | --- |
| C0168T22 | Infliximab 3 mg/4week | FDA | 0.86 | 0.422 | In FDA report, analysis reflects actual treatment received rather than the treatment group to which they were randomly assigned. Two placebo patients received 0.5 mg/kg INF for one infusion each and were included in the 3mg/kg q 8 weeks treatment group. One patient in the 10 mg/kg q4 weeks group received 6 treatments, which was more in accordance with the 3 mg/kg q 8 week group. | Patient inclusion |
|  | Infliximab 3 mg/8week | FDA | 0.94 | 0.779 |  |  |
|  | Infliximab 10 mg/4week | FDA | 0.98 | 0.915 |  |  |
|  | Infliximab 10 mg/8week | FDA | 0.99 | 0.954 |  |  |
| WA17822 | Tocilizumab 4 mg | Journal | 1.21 | 0.320 | 4 patients in the placebo group withdrew due to AEs in the journal report vs. 5 in the FDA report. | Counting discrepancy |
|  | Tocilizumab 8 mg | Journal | 1.21 | 0.331 |  |  |
| WA17824 | Tocilizumab 8 mg | Journal | 1.87 | 0.004 | Discrepancies between journal report and FDA for two arms. For 8 mg and placebo MTX: 11/288 and 15/284 patients withdrew due to AEs in the journal report vs. 8/286 and 6/284 in the FDA report, respectively. | Counting discrepancy |
| WA18062 | Tocilizumab 4 mg | Journal | 1.15 | 0.437 | In Table 1, number of patients agrees with ITT in FDA report; however, in Table 3, numbers reflect randomized patients and therefore do not agree with the Methods section which uses the ITT approach. | Analytic approach |
|  | Tocilizumab 8 mg | Journal | 1.30 | 0.145 |  |  |
| WA18063 | Tocilizumab 8 mg | Journal | 1.10 | 0.515 | Discrepancies between journal and FDA reports in both arms. For 8 mg and placebo: 30/803 and 8/414 patients withdrew due to AEs in journal report vs. 30/803 and 7/413 in FDA report, respectively. | Counting discrepancy |

RCT-randomized controlled trial, No.-number of patients, WdAEs - withdrawal of patients due to adverse events response measure, FDA - Food and Drug Administration
